# Cost of illness of patients with small fiber neuropathy in the Netherlands

**DOI:** 10.1097/j.pain.0000000000003008

**Published:** 2023-08-04

**Authors:** Margot Geerts, Janneke G.J. Hoeijmakers, Yvonne van Eijk-Hustings, Lloyd Brandts, Carla M.L. Gorissen-Brouwers, Ingemar S.J. Merkies, Manuela A. Joore, Catharina G. Faber

**Affiliations:** a Department of Neurology, Maastricht University Medical Center+, School of Mental Health and Neuroscience, Maastricht, the Netherlands; b Department of Clinical Epidemiology and Medical Technology Assessment, Maastricht University Medical Center+, CAPHRI Care and Public Health Research Institute, Maastricht, the Netherlands; c Department of Neurology, Curacao Medical Center, J. H. J. Hamelbergweg, Willemstad, Curacao

**Keywords:** Small fiber neuropathy, Cost of illness study, Neuropathic pain, Health-related quality of life, Health care and societal perspective

## Abstract

Neuropathic pain is associated with substantial healthcare costs. However, cost-of-illness studies of small fiber neuropathy (SFN) are scarce. Our aim was to estimate the healthcare, patient and family, and productivity costs of patients with SFN in the Netherlands from a healthcare and societal perspective. In addition, the association of costs with age, pain impact on daily life, anxiety, depression, and quality of life (Qol) were examined. Cost questionnaires were completed by 156 patients with confirmed SFN. The average annual total health care and societal cost (€, 2020) was calculated at patient, SFN adult population, and societal level. The average annual healthcare, patient and family, and productivity costs per patient with a Pain Impact Numerical Rating Scale of 0 to 3 (mild), 4 to 6 (moderate), and 7 to 10 (severe) were calculated by using the cost questionnaire data. Quality of life was determined by the EuroQol 5D utility scores. Anxiety and depression were assessed using the Hospital Anxiety and Depression Scale. Associations of all costs were analyzed using linear regression analyses. At the patient level, the average annual SFN healthcare and societal cost of SFN was €3614 (95% confidence interval [CI] €3171-€4072) and €17,871 (95% CI €14,395-€21,480). At the SFN population level, the average healthcare costs were €29.8 (CI €26.4-€34.2) million, and on a societal level, these were €147.7 (CI 120.5-176.3) million. Severe pain was associated with significant lower Qol and higher depression scores, higher healthcare, patient and family, and productivity costs (*P* < 0.001).

## Abstract 2

Small fiber neuropathy societal costs were estimated at €148 million with daily practice small fiber neuropathy patient data. Health-related quality of life was the key association on health care and societal costs.

## 1. Introduction

Small fiber neuropathy (SFN), with a prevalence rate of 53 per 100,000 inhabitants,^25^ is a disorder of the thinly myelinated Aδ fibers and unmyelinated C fibers and clinically dominated by neuropathic pain and autonomic complaints.^12,29^ The diagnosis of SFN is based on at least 2 small nerve fiber–related clinical signs of the patient, normal nerve conduction studies (NCS) and an abnormal intraepidermal nerve fiber density (IENFD) in skin biopsy, and/or abnormal quantitative sensory testing (QST).^11,17^ Diabetes mellitus, autoimmune diseases, and sodium channel gene mutations are the most common conditions observed in patients with SFN, but in 53% of cases, no underlying condition is found.^8^ In addition to the initial treatment of the underlying condition, neuropathic pain treatment is needed,^15^ but generally, these yield disappointing results.^15,29^ Severe SFN leads to a reduced quality of life (Qol),^2^ with commonly associated anxiety and depression greatly interfering with patients' ability to function.^18^ Higher age and a higher number of comorbidities are prognostic factors for higher health care and productivity costs.^9,32^

The US annual total health care and patient and family costs of idiopathic painful neuropathy with SFN involvement since 2012 were estimated at $8055 (€7403) per patient.^27^ Total costs of work productivity loss were estimated to be $13,733 (€12,621) per patient due to a poorer health status, worse sleep outcomes, and loss of productivity.^27^ A significant association was found between health care and patient and family costs and pain severity, but no statistically significant associations were found in productivity costs.^27^ However, because of the differences between the United States and the Netherlands in how painful idiopathic SFN is diagnosed, the fact that fewer patients in the United States were in a paid employment and the differences between the healthcare system of the United States and the Netherlands, the study populations and the costs do not lend themselves for straightforward comparison. A cost of illness (COI) study of confirmed SFN has not yet been conducted. Therefore, the costs of SFN and the factors which influence healthcare consumption and productivity costs remain largely unknown. This COI study aims to examine the healthcare, patient and family, and productivity costs of patients with confirmed (by skin biopsy or QST proven) SFN in the Netherlands to estimate the annual SFN costs from a healthcare and a societal perspective. In addition, the associations of age, pain impact on daily life, anxiety, depression, and health-related Qol on these costs were investigated.

## 2. Methods

### 2.1. Study design and patients

This COI study was conducted at the diagnostic SFN service of the SFN Center of the Maastricht University Medical Center+ in Maastricht, the Netherlands. The SFN Center is a tertiary referral center for patients with suspected SFN, evaluating approximately 500 patients yearly. The diagnostic SFN service is based on a 1-day stay at the neurological day care unit with time slots reserved for interviewing, examining, diagnostic tests, and analyzing and discussing the findings among a multidisciplinary team. Diagnostic tests include a skin biopsy for identifying abnormalities in IENFD, NCS, and QST.

### 2.2. Patient population and selection

**Figure 1.**
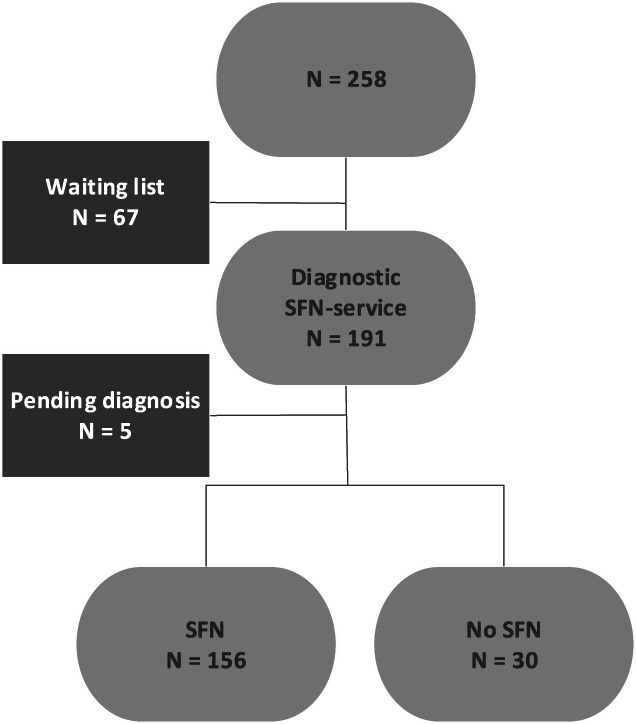
Study flowchart at the time of data analysis on August 21th 2020. SFN, small fiber neuropathy.

Using the waiting list registration, all patients with suspected SFN and ≥18 years of age, referred to the SFN Center between April 2017 and February 2020, were invited by e-mail to participate in this study. They were given access to an Internet-based electronic environment to complete the online questionnaires. For those patients not able to complete the online questionnaire, a paper version was provided. All questionnaires were completed before the patients' visit to the SFN Center, so while on the waiting list. Exclusion criteria were declining participation and significant language barrier. Only data of patients with confirmed SFN were analyzed. Between April 2017 and February 2020, 258 patients participated in the study. The flowchart of the waiting list up to and including their visit to the diagnostic SFN service is shown in Figure 1, and 67 patients did not visit the SFN Center yet. In 81.7% of the 191 patients, the diagnosis of SFN was confirmed (n = 156); in 15.7%, the diagnosis could not be confirmed, and these were omitted from further analysis in this study.

### 2.3. Standard protocol approvals, registrations, and patient consents

The study was approved by the Medical Ethics Committee of Maastricht UMC+ (15-4-004). Informed consent of all patients was obtained before participating in the study, according to the principles of the Declaration of Helsinki.^35^

### 2.4. Data collection

Sociodemographic data (eg, age, sex, and education) and data on clinical characteristics (duration of SFN complaints) and patient-related outcome measures (PROMs; eg, pain impact on daily life, anxiety, depression, and health-related QoL) were obtained by the online survey and the electronic patient file.

Pain impact on daily life was measured using the 11-point Pain Impact Numerical Rating Scale (Pain Impact NRS, with 0 meaning having no impact and 10 meaning having the worst imaginable impact).^14^

Anxiety and depression were assessed using the Hospital Anxiety and Depression Scale (HADS) questionnaire, disaggregated for anxiety (HADS-A) and depression (HADS-D). Each subscale consists of 7 questions with answers recorded on a 4-point Likert scale. Scores can range from 0 to 21. Higher scores indicate more symptoms of anxiety and depression.^22^

The 5-level EuroQol 5D (EQ-5D-5L) was used to measure generic health-related QoL.^20^ The EQ-5D-5L consists of a Visual Analog Scale (VAS), which ranges from worst (0) to the best imaginable health (100), and 5 additional questions, each representing a health-related QoL dimension. These 5 additional questions covered mobility, self-care, usual activity, pain/discomfort, and anxiety/depression.^20^ Each question has 5 response levels, classifying the severity of complaints for that specific dimension and allowing 3125 possible state-of-health combinations. These were converted into EQ-5D utility scores according to the Dutch tariff.^33^ Possible EQ-5D utility scores range from −0.446 to 1.00,^33^ with −0.446 being the worst imaginable state of health and 1.00 the best.

Healthcare and patient and family costs related to SFN were measured with the iMTA Medical Consumption Questionnaire. Participants were asked to only report resource use and costs related to neuropathic and autonomic complaints of SFN, within a 3-month recall period.^4^ The iMTA Medical Consumption Questionnaire includes questions on the utilization of general practitioner (GP) visits, medical specialist visits, other healthcare provider visits (eg, psychologist), emergency room (ER) visits, hospital outpatient visits, and hospitalizations and use of paramedical care, prescription medications, outpatient tests, and procedures. In addition, questions on out-of-pocket costs for medical care and nonmedical resources (help with household or garden work, travel expenses, and help with daily activities, such as cooking) related to SFN are also included.

The iMTA Productivity Cost Questionnaire (iPCQ) was used to measure productivity costs.^5^ Participants were asked to only report data on paid employment, reduced work schedule, absenteeism, unemployment, and the costs of productivity loss for unpaid employment activities as a result of neuropathic and autonomic complaints of SFN, using a 3-month recall period, in accordance with the Dutch guideline for cost research*.*^19^

Subjects were asked to score the pain impact on their working productivity of the last week on the Pain Impact NRS.^14^

### 2.5. Statistical analysis

Descriptive statistics were used to present sociodemographic, clinical characteristics, and PROMs data. The Pain Impact NRS scores on daily life were used to categorize subjects into 1 of 3 pain impact groups based on established cut-off points for neuropathic pain (0-3, mild; 4-6, moderate; and 7-10, severe).^36^ Differences in demographics, pain impact on daily life, anxiety, depression, and health-related Qol among these groups were tested with an analysis of variance (Kruskal–Wallis test) where appropriate for the continuous variables, whereas a Chi-square test was used for the categorical variables. The continuous variables were tested for normality using a Kolmogorov-Smirnov test.

Healthcare, patient, and family costs were calculated using per unit costs obtained from the Dutch guideline for cost research.^30^ Unit costs were converted to the reference year 2020 by means of index numbers.^6^ To acquire annual average overall costs per patient, the measured 3-month costs were multiplied by 4.

The productivity costs of paid employment were quantified using the friction cost approach, in which productivity loss is restricted to 85 calendar days (12 weeks).^19^ The cost of an hour of productivity loss from paid employment was calculated by using the Dutch guideline for cost research based on the average hourly salary costs per paid worker.^19^

Productivity costs from unpaid employment were valued on the basis of replacement costs for household care. This was equated to a standard hourly rate for cleaning work, as used by the Dutch Central Administration Office (CAK).^19^

The total societal costs consist of the sum of healthcare, patient and family, and productivity costs. Average total societal costs per patient were multiplied by the prevalence of SFN for the general adult population to estimate the total COI of SFN for the Dutch society. In 2020, the Dutch adult population aged ≥20 years totaled 15,592,909 residents (Central Bureau of Statistics 2020). By applying the prevalence rate of 53 cases per 100,000 inhabitants, it was calculated that approximately 8264 adults in the Netherlands have SFN.

Usually, cost data are not normally distributed. Therefore, a nonparametric bootstrap resampling procedure with 1000 simulations was performed in SPSS to determine statistical uncertainty of the cost estimates per category. The differences in costs among the pain impact groups were established by calculating confidence intervals (CI) by the bootstrapping procedure.

Multivariate linear regression analyses were performed to estimate the association of age, pain impact on daily life, health-related Qol, anxiety and depression, with healthcare, patient and family, productivity, and societal costs. These variables were selected based on their relevance according to the literature.^2,18,32^ A backward stepwise method was used to test for interaction between the independent variables on all outcomes using an α of 0.05. For statistically significant interactions, results were presented with an interaction term whenever there was at least one continuous variable or stratified per category whenever there was at least one categorical variable. A log transformation was performed on the dependent variables to resemble normality. Where possible, the observed β′s were back transformed into a relative difference (in %) using the formula: (exp[β] − 1) × 100%. All analyses were performed using IBM Statistics SPSS version 25.0.

## 3. Results

### 3.1. Demographic and clinical characteristics

Demographics, clinical characteristics, and PROMs of the 156 patients with confirmed SFN are shown in Table 1. The median age was 55.2 (IQR 47.2–61.6) years, and the majority (66.7%) was female.

**Table 1:** Baseline characteristics of the SFN study population.

	Overall (N = 156)	Mild (N = 33)	Moderate (N = 36)	Severe (N = 87)	*P*
Age (years)					
Average (CI)	53.8 (51.8-55.6)	55.8 (51.0-60.0)	54.9 (51.5-58.4)	53.0 (50.7-55.6)	0.459
Median (IQR)	55.2 (47.2-61.6)	56.8 (45.3-65.0)	55.5 (50.3-61.0)	53.8 (45.0-61.4)	0.640
Sex, n (%)					0.567
Male	52 (33.3)	13 (43.3)	13 (36.1)	26 (29.9)	
Pain impact NRS on daily life, median (IQR)	7.0 (4.0-8.0)	2.0 (0.0-3.0)	5.0 (4.0-6.0)	8.0 (7.0-8.0)	<0.001*
HADS, median (IQR)					
Anxiety	7.0 (5.0-10.0)	7.0 (4.9-7.5)	7.0 (6.0-8.4)	7.0 (7.0-8.7)	0.129
Depression	7.0 (4.0-9.0)	4.0 (3.7-5.8)	7.0 (5.3-7.4)	7.0 (6.9-8.5)	<0.001†
EQ-5D-5L utility score, median (IQR)	0.59 (0.42-0.71)	0.79 (0.71-0.84)	0.67 (0.57-0.72)	0.46 (0.36-0.61)	<0.001*
Level of education, n (%)					0.176
Low	21 (13.5)	1 (3.0)	5 (13.9)	15 (17.3)	
Medium	71 (45.5)	14 (42.4)	16 (44.4)	41 (47.1)	
High	61 (39.1)	15 (45.5)	15 (41.7)	31 (35.6)	
Missing	3 (1.9)	3 (9.1)			
Duration SFN complaints in years before diagnosis, median (IQR)	5.0 (2.0-9.3)	4.0 (2.0-10.0	4.0 (3.0-7.8)	5.0 (3.0-10.0)	0.421
Diagnostic tests, n (%)					0.909
Abnormal IENFD and abnormal TTT	93 (59.6)	19 (57.6)	20 (55.6)	54 (62.1)	
Abnormal IENFD	29 (18.6)	7 (21.2)	8 (22.2)	14 (16.1)	
Abnormal TTT	34 (21.8)	7 (21.2)	8 (22.2)	19 (21.8)	
Cause of SFN, n (%)					0.056
Idiopathic	96 (61.5)	24 (72.7)	27 (75.0)	45 (51.7)	
Glucose intolerance	7 (4.5)	2 (6.1)	1 (2.8)	4 (4.6)	
Diabetes mellitus	3 (1.9)	0 (0.0)	0 (0.0)	3 (3.5)	
Vitamin B12 deficiency	9 (5.8)	0 (0.0)	1 (2.8)	8 (9.2)	
Sodium channel gene mutations	8 (5.1)	1 (3.0)	0 (0.0)	7 (8.0)	
Immunological abnormalities	28 (18.0)	6 (18.2)	7 (19.4)	15 (17.2)	
Chemotherapy	4 (2.6)	0 (0.0)	0 (0.0)	4 (4.6)	
Monoclonal gammopathy of undetermined significance	1 (0.6)	0 (0.0)	0 (0.0)	1 (1.2)	

* Significantly different distributed in all pain groups.

† Significantly different distributed in the mild pain and severe pain group.

CI, confidence interval; IQR, interquartile range; Pain Impact NRS, Numerical Rating Scale; HADS, Hospital Anxiety and Depression Scale; EQ-5D-5L, EuroQol 5D Questionnaire; SFN, small fiber neuropathy.

### 3.2. Differences between the 3 pain impact groups

Between all 3 pain impact groups, statistically significant differences were seen in the Pain Impact NRS on daily life and health-related QoL utility scores (*P* < 0.001). Furthermore, statistically significant higher depression scores were observed in the severe pain group compared with the mild pain impact group (*P* < 0.001). No statistically significant differences were seen between the 3 pain impact groups regarding age, sex, level of education, duration of SFN complaints, anxiety, and diagnostic tests.

### 3.3. Healthcare costs

The annual average SFN healthcare costs are presented in Table 2. Sixty-five percent of the patients visited a GP, with a total annual average GP costs per person of €292 (95% CI €236,8-€348,3). Medical specialists were visited by 87% of the patients; the most frequently visited medical specialists were the neurologist (83.3%), the internist (14.1%), and the anesthesiologist (12.2%). Active medical pain treatment was used by 73% of the patients. The most frequently used active medical pain treatments were antiepileptics (32%), tricyclic antidepressants (28%), and opioids (14%). The total annual average healthcare costs per person were €3614 (95% CI €3171-€4072), with medical specialist care and active medical treatment being the largest contributors (€1,305, 95% CI €1144-€1,485, and €989, 95% CI €760-€1225 per patient, respectively).

**Table 2:** Annual SFN-related healthcare costs.

	Unit costs in €	Total N = 156		Mild subgroup (pain impact NRS: 0-3) N = 33		Moderate subgroup (pain impact NRS: 4-6) N = 36		Severe subgroup (pain impact NRS: 7-10) N = 87		*P*
		Annual contacts average (CI)	Annual costs average in € (CI)	Annual contacts average (CI)	Annual costs average in € (CI)	Annual contacts average (CI)	Annual costs average in € (CI)	Annual contacts average (CI)	Annual costs average in € (CI)	
GP practice	34.55	10.1 (8.3-12.2)	291.6 (236.8-348.3)	4.6 (2.4-7.3)	143.0 (77.8-216.9)	9.2 (5.5-13.1)	264.7 (166.5-362.3)	12.6 (9.8-15.8)	359.2 (277.9-441.6)	<0.05*
GP evening and weekends	86.36	0.2 (0.0-0.4)	6.6 (0.0-17.7)	0.0 (0.0-0.0)	0.0 (0.0-0.0)	0.2 (0.0-0.7)	9.6 (0.0-31.4)	0.2 (0.0-0.5)	7.9 (0.0-26.9)	0.590
Emergency room	271.15	0.2 (0.1-0.4)	62.6 (20.9-111.2)	0.1 (0.0-0.4)	32.9 (0.0-108.5)	0.1 (0.0-0.4)	30.1 (0.0-108.5)	0.3 (0.1-0.6)	87.3 (14.1-178.3)	0.684
Medical specialists	101.81	12.8 (11.2-14.5)	1305.3 (1143.5-1485.3)	9.5 (6.3-13.6)	962.6 (667.4-1340.8)	12.6 (9.2-16.4)	1278.3 (935.2-1675.2)	14.2 (12.0-16.8)	1446.4 (1226.2-1705.7)	<0.05*
Neurologist	101.81	7.2 (6.3-8.0)	728.3 (652.6-819.7)	6.5 (5.1-8.1)	666.4 (509.1-839.2)	6.6 (5.0-8.4)	667.4 (502.3-837.7)	7.6 (6.4-8.9)	777.0 (654.5-989.9)	0.502
Internist	101.81	1.0 (0.6-1.4)	101.8 (54.8-151.4)	0.1 (0.0-0.4)	12.3 (0.0-40.7)	0.9 (0.2-2.0)	90.5 (19.9-203.5)	1.4 (0.7-2.1)	140.4 (69.4-220.9)	0.089
Anesthesiologist	101.81	0.9 (0.5-1.5)	94.0 (47.0-151.3)	0.0 (0.0-0.0)	0.0 (0.0-0.0)	1.0 (0.2-2.0)	101.8 (21.4-214.9)	1.2 (0.5-2.2)	126.4 (56.8-217.8)	0.054
Rheumatologist	101.81	0.3 (0.1-0.5)	28.7 (13.1-47.0)	0.1 (0.0-0.4)	12.3 (0.0-40.7)	0.1 (0.0-0.4)	11.3 (0.0-38.2)	0.4 (0.2-0.7)	42.1 (19.2-70.4)	0.198
Cardiologist	101.81	0.2 (0.1-0.3)	18.3 (5.2-33.9)	0.2 (0.0-0.6)	24.7 (0.0-62.6)	0.0 (0.0-0.0)	0.0 (0.0-0.0)	0.2 (0.0-0.5)	23.4 (4.8-46.8)	0.335
Hospitalization	501.12	0.2 (0.0-0.3)	77.1 (25.7-154.2)	0.0 (0.0-0.0)	0.0 (0.0-0.0)	0.2 (0.0-0.5)	111.4 (0.0-286.4)	0.2 (0.0-0.5)	92.2 (0.0-217.3)	0.423
Physiotherapy	34.55	12.3 (8.6-16.4)	424.3 (306.5-559.0)	7.4 (1.8-14.7)	255.5 (67.0-518.3)	8.8 (4.2-14.2)	303.3 (138.2-494.7)	15.6 (10.1-21.9)	538.5 (351.6-745.6)	0.179
Psychologist or psychiatrist	82.7	2.2 (1.3-3.3)	178.1 (103.9-265.0)	0.2 (0.0-0.6)	20.0 (0.0-53.4)	3.4 (1.4-6.2)	284.9 (104.5-496.0)	2.3 (1.0-3.9)	193.9 (88.0-318.5)	0.077
Other healthcare providers	65.32	4.3 (2.8-6.0)	279.7 (182.6-385.2)	1.7 (0.4-3.3)	110.8 (27.1-206.8)	5.8 (1.8-10.5)	377.4 (130.7-718.2)	4.6 (2.7-7.0)	303.3 (176.5-434.3)	0.568
Active pain treatment	Various	5.8 (4.9-6.6)	988.8 (760.4-1224.8)	2.5 (1.4-4.1)	236.5 (105.6-380.0)	5.3 (3.7-7.1)	724.7 (386.3-1124.9)	7.2 (5.8-8.4)	1383.4 (1035.0-1759.3)	<0.001*
TCA	37.7	1.3 (0.9-1.7)	49.3 (36.7-62.8)	1.2 (0.3-2.3)	45.7 (10.8-93.1)	1.2 (0.7-1.9)	46.1 (22.9-69.6)	1.4 (0.9-1.8)	52.0 (35.3-69.7)	0.447
SNRI/SSRI	38.3	0.5 (0.3-0.8)	19.6 (9.8-32.4)	0.1 (0.0-0.4)	4.6 (0.0-16.4)	1.1 (0.3-2.2)	42.6 (11.4-88.7)	0.4 (0.2-0.7)	15.8 (7.1-26.1)	0.161
Antiepileptics	227.2	1.5 (1.2-1.9)	343.7 (262.2-431.1)	0.6 (0.1-1.2)	137.7 (32.5-259.7)	1.1 (0.4-2.0)	252.4 (95.7-426.0)	2.0 (1.5-2.6)	459.6 (334.3-589.2)	<0.05*
Acetaminophen/NSAIDs	44.7	0.6 (0.4-0.8)	26.4 (16.0-37.8)	0.0 (0.0-0.0)	0.0 (0.0-0.0)	0.6 (0.1-1.0)	24.8 (5.8-49.3)	0.8 (0.5-1.2)	37.0 (20.1-56.4)	<0.05*
Opioids/opiates	528.6	0.8 (0.5-1.1)	420.2 (257.5-596.4)	0.0 (0.0-0.0)	0.0 (0.0-0.0)	0.7 (0.1-1.3)	352.4 (66.1-684.1)	1.1 (0.7-1.7)	607.6 (368.8-906.1)	<0.05*
Total direct costs	Various	—	3614.2 (3170.8-4071.5)	—	1761.4 (1310.6-2278.4)	—	3384.2 (2516.0-4272.9)	—	4412.1 (3815.8-5123.7)	<0.001†

GP practice includes GP visits, GP home visit, and GP telephone calls.

* Significantly different distributed in all pain groups.

† Significantly different distributed in the mild and moderate pain group and in the mild pain and severe pain group.

€, Euro; CI, Confidence Interval; Pain Impact NRS, Numeric Rating Scale; TCA, Tricyclic Antidepressants; SNRI, Selective Serotonin and Noradrenalin Reuptake Inhibitor; SSRI, Selective Serotonin Reuptake Inhibitor.

### 3.4. Patient and family costs

The total annual average SFN patient and family costs was €2076 (95% CI €1032-€3759) per patient (Table 3). Personal care was only received by patients in the severe pain impact group, with an annual average of €246 (95% CI €0.0-€705) per patient. Domestic and private paid domestic help only occurred in the moderate and severe pain impact group, with an annual average cost of €532 (95% CI €118-€1219) and €1178 (95% CI €56-€184) per patient, respectively. Half of the patients used informal care, which accounted for >80% of the total patient and family costs (annual average costs per patient €1,739, 95% CI €1181-€2386). The highest travel expenses were found in the severe pain impact group, with an annual average of €253 (95% CI €75-€612) per patient. Over-the-counter medication was used by 44% of the patients, and more than a quarter of the patients bought medical devices, on which an annual average of €550 (95% CI €358-€786) per patient was spent.

**Table 3:** Annual SFN-related patient and family costs.

	Unit costs in €	Total N = 156		Mild subgroup (pain impact NRS: 0-3) N = 33		Moderate subgroup (pain impact NRS: 4-6) N = 36		Severe subgroup (pain impact NRS: 7-10) N = 87		*P*
		Annual contacts average (CI)	Annual costs average (CI) in €	Annual contacts average (CI)	Annual costs average (CI) in €	Annual contacts average (CI)	Annual costs average (CI) in €	Annual contacts average (CI)	Annual costs average (CI) in €	
Personal care or domestic help by home nursing organization	32.45	12.6 (2.9-28.4)	407.7 (71.6-864.4)	0.0 (0.0-0.0)	0.0 (0.0-0.0)	5.1 (0.0-15.2)	165.9 (0.0-457.4)	20.4 (2.2-48.3)	662.4 (86.1-1421.7)	0.132
Personal care	52.35	4.7 (0.0-13.4)	245.6 (0.0-704.7)	0.0 (0.0-0.0)	0.0 (0.0-0.0)	0.0 (0.0-0.0)	0.0 (0.0-0.0)	8.4 (0.0-24.3)	440.5 (0.0-1292.4)	0.300
Domestic help	24.01	22.2 (4.9-49.4)	531.9 (118.2-1219.4)	0.0 (0.0-0.0)	0.0 (0.0-0.0)	13.3 (0.0-35.7)	320.1 (0.0-913.5)	34.2 (6.0-85.1)	821.3 (134.0-2062.5)	0.241
Private paid domestic help	Variable	5.7 (3.2-8.3)	117.8 (56.2-183.5)	0.0 (0.0-0.0)	0.0 (0.0-0.0)	10.0 (4.3-16.8)	261.6 (98.3-442.4)	6.0 (2.6-9.9)	104.1 (37.7-190.1)	<0.05‡
Informal care by the family of friends	14.66	118.6 (79.8-163.8)	1738.6 (1181.4-2385.9)	73.9 (1.4-185.4)	1083.9 (18.8-2726.8)	70.9 (24.2-130.2)	1039.2 (342.4-1947.1)	154.3 (102.2-219.9)	2261.3 (1520.5-3213.1)	<0.001*
Travel expenses	Various	—	187.5 (77.2-367.1)	—	135.0 (54.0-306.8)	—	77.7 (61.0-97.7)	—	252.8 (75.3-612.1)	<0.05‡
Over-the-counter medication	Various	3.1 (2.4-3.8)	39.1 (27.9-50.8)	2.2 (1.0-3.5)	27.4 (8.7-49.6)	2.1 (1.1-3.3)	29.4 (10.3-51.9)	3.8 (2.8-4.9)	47.4 (32.6-63.9)	0.140
Medical devices	Various	—	549.5 (358.2-786.4)	—	91.0 (12.1-204.2)	—	341.1 (92.0-679.9)	—	809.8 (485.5-1189.6)	<0.05*
Other costs	Various	—	846.1 (295.0-1882.9)	—	124.4 (27.4-253.0)	—	313.9 (29.7-704.8)	—	1340.1 (357.6-3085.4)	0.426
Total costs	Various	—	2075.9 (1031.8-3758.5)	—	253.4 (101.1-445.7)	—	1187.7 (493.4-2293.6)	—	3134.7 (1359.6-5689.2)	≤0.001*

* Significantly different distributed in all pain groups.

†Significantly different distributed in the moderate and severe pain group.

‡ Significantly different distributed in the mild pain and severe pain group.

€, Euro; CI, Confidence Interval; Pain Impact NRS, Numerical Rating Scale.

### 3.5. Costs of productivity loss

Less than half of the patients were in part-time paid employment (25%) or full-time paid employment (22%), and 21% was disabled. The average SFN productivity costs are presented in Table 4. Among patients in paid employment, the average weekly contract hours were 30.8 hours (95% CI 27.7-32.4), with an average monthly net income of €1387 (95% CI €1087-€1681) based on patients' reported net incomes. Absenteeism in the last quarter occurred in 56% of the patients in paid employment, with an average of 22.9 days (95% CI 16.7-29.1). Costs of productivity loss due to absenteeism per patient in paid employment was €3540 (95% CI €2486-€4676) per quarter. 72% of all patients reported a reduction in performing daily household activities due to SFN, with an average quarterly reduction of 517.4 hours (95% CI 388.9-679.4) per patient. Average costs of productivity loss because of limitations in performing daily household tasks due to SFN was €8045 (95% CI €5978-€10,255) per patient. The total average quarterly costs of productivity loss of all patients were €12,167 (95% CI €13,351-€21,926) per patient.

**Table 4:** Annual SFN-related productivity loss.

	Overall N = 156	Mild subgroup (pain impact NRS: 0-3) N = 33	Moderate subgroup (pain impact NRS: 4-6) N = 36	Severe subgroup (pain impact NRS: 7-10) N = 87	*P*
Contract hours per week, average (CI)	30.8 (27.7-32.4)	35.3 (31.5-38.8)	22.1 (17.7-27.3)	31.4 (28.5-34.5)	0.410
Working days per week, average (CI)	4.3 (4.1-4.6)	4.5 (4.0-4.8)	3.9 (3.2-4.8)	4.4 (4.2-4.7)	0.785
Net income per month in €, average (CI)	1387.1 (1087.3-1681.3)	1505.3 (810.4-2133.9)	1349.0 (724.2-2025.7)	1356.1 (938.0-1743.4)	0.905
Less working hours due to neuropathy (yes/no), n (%)	39/117 (25.0/75.0)	3/30 (9.1/90.9)	10/26 (27.8/72.2)	26/61 (29.9/70.1)	0.058
Absenteeism in the last quarter (yes/no), n (%)	41/32 (56.2/43.8)	3/30 (9.1/90.9)	8/28 (22.2/77.8)	30/57 (34.5/65.5)	<0.05*
Average total days (CI)	22.9 (16.7-29.1)	6.9 (0.0-17.7)	14.2 (5.6-23.7)	33.0 (23.9-43.0)	<0.05*
Average frequency (CI)	0.7 (0.5-0.8)	0.2 (0.0-0.4)	0.5 (0.2-0.8)	0.9 (0.7-1.2)	<0.05*
Absenteeism due to neuropathy at this moment (yes/no), n (%)	32/41 (43.8/56.2)	2/14 (12.5/87.5)	6/11 (35.3/64.7)	24/16 (60.0/40.0)	<0.05*
Costs of productivity loss due to absenteeism in €, average (CI)	3539.5 (2485.7-4676.2)	1108.0 (0.0-2891.6)	2210.8 (723.1-3961.7)	5014.3 (3260.4-6882.0)	<0.05*
Being limited in performing daily household tasks (yes/no), n (%)	113/43 (27.6/72.4)	9/24 (27.3/72.7)	25/11 (69.4/30.6)	79/8 (90.8/9.2)	<0.001†
Average total hours (CI)	517.4 (388.9-679.4)	90.1 (9.8-217.5)	431.8 (236.8-666.5)	717.2 (503.5-956.7)	<0.001*
Costs of productivity loss because of limitations in performing daily household tasks in €, average (CI)	8604.3 (6356.4-11,189.2)	1497.7 (164.3-3444.7)	7180.5 (3927.0-11,297.6)	11,927.2 (8464.7-16,007.4)	<0.001*
Total costs of productivity loss in €, average (CI)	12,166.6 (13,351.2-21,925.5)	2598.5 (678.7-4971.8)	9391.2 (5121.0-14,740.9)	16,999.8 (13,023.8-21,432.6)	<0.001*
Societal costs in €, average (CI)	17,871.2 (14,395.0-21,480.7)	4613.3 (1896.4-7330.2)	13,963.2 (8679.9-19,246.4)	24,594.4 (19,350.4-29,838.4)	<0.001*

† Significantly different distributed in all pain groups.

* Significantly different distributed in the mild pain and severe pain group.

€, Euro; CI, confidence interval; Pain Impact NRS, Numerical Rating Scale.

### 3.6. Societal costs

**Figure 2.**
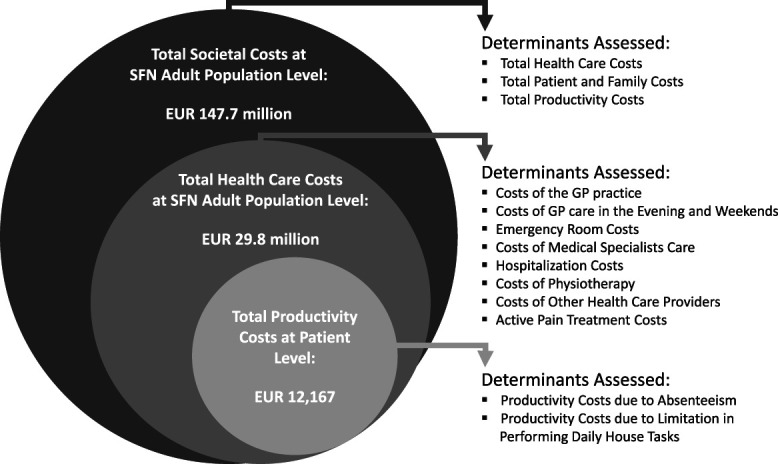
Cost of illness overview of SFN in the Netherlands (2020).

The COI of patients with SFN (€, 2020) in the Netherlands is presented in Figure 2 and will be discussed hereafter. The total average SFN productivity costs accounted for 68% of the total societal costs at the patient level. The total healthcare costs of the adult population with SFN were estimated to be €29.8 million (95% CI: €26.5 million-€33.7 million). Total average societal costs of the adult general population with SFN in the Netherlands were estimated to be €147.7 million (95% CI €120.5 million-€176.3 million).

### 3.7. Statistically significant associations with costs

There were no statistically significant associations found between age, pain impact on daily life, health-related Qol, anxiety and depression, and SFN healthcare costs (Table 5). In the SFN patient and family costs, a statistically significant interaction was found between health-related QoL and anxiety (p interaction ≤ 0.001). Therefore, results for patients with mild/moderate anxiety (symptoms ≤ 10) and severe anxiety (symptoms ≥ 11) are presented separately in Table 5. Health-related QoL was statistically significant inversely associated with patient and family costs in patients with mild or moderate anxiety symptoms (*P* < 0.01). An increase of 0.1 point on the EQ-5D utility score was associated with a 13.2% decrease (95% CI −20.8 to 4.9) in patient and family costs. No significant association with health-related QoL was observed in patients with severe anxiety symptoms (4.2% increase, 95% CI −10.0 to 20.6, *P*-value 0.571). For the costs of productivity loss and societal costs, a significant interaction was observed between pain (pain impact NRS ≤ 6 vs pain impact NRS ≥ 7) and health-related QoL (per 0.1 point increase, continuous) (*P* < 0.05), and therefore, these results are presented separately by the pain group in Table 5. Health-related QoL was statistically significant inversely associated with productivity costs in the pain impact group NRS ≤ 6 (*P* ≤ 0.001). An increase of 0.1 point on the EQ-5D utility score was associated with a 41.4% decrease (95% CI: −57.5 to −19.3) in productivity costs. No statistically significant association with health-related QoL was observed in patients with a pain impact NRS of ≥ 7 (3.9% decrease, 95% CI −17.7% to 12.3%, *P*-value 0.612). Regarding the societal costs, health-related QoL was statistically significant inversely associated in the pain impact groups NRS ≤ 6 and NRS ≥ 7 (*P* < 0.01 vs *P* < 0.05, respectively). A 0.1 point increase on the EQ-5D utility score was associated with a decrease of 14.6% (−23.7% to −4.5%) in societal costs in the pain impact group NRS ≤ 6 and a decrease of 5.8% (−10.8% to −0.5%) in societal costs in the pain impact group NRS ≥ 7.

**Table 5:** Results of multivariate regression analysis with a backward stepwise method.

Dependent variable	n	Independent variable	B (95% CI)	% (95% CI)	Standardized B	*P*
Health care costs	156	Age	−0.007 (−0.016 to 0.001)	−0.7 (−1.6 to 0.1)	−0.127	0.096
		Health-related QoL (EQ-5d-5L) per 0.1 point increase	−0.053 (−0.117 to 0.011)	−5.16 (−11.0 to 1.1)	−0.160	0.101
		PI-NRS	0.044 (−0.002 to 0.090)	4.5 (−0.199 to 9.4)	0.179	0.058
		HADS_anxiety	0.024 (−0.005 to 0.054)	2.4 (−0.50 to 5.55)	0.142	0.107
		HADS_depression	−0.001 (−0.035 to 0.032)	−0.1 (−3.44 to 3.25)	−0.006	0.946
Patient and family costs	156					
Anxiety group (A ≤ 10)	124	Age	−0.003 (−0.014 to 0.009)	−0.3 (−1.0 to 0.9)	−0.035	0.679
		Health-related QoL (EQ-5d-5L) per 0.1 point increase	−0.141 (−0.233 to −0.050)	−13.2 (−20.8 to 4.9)	−0.333	0.003
		PI-NRS	0.052 (−0.012 to 0.117)	5.3 (−1.2 to 12.4)	0.169	0.112
		HADS_depression	−0.025 (−0.069 to 0.020)	−2.4 (−6.7 to 2.0)	−0.101	0.277
Anxiety group (A ≥ 11)	32	Age	−0.005 (−0.026 to 0.017)	−0.5 (−2.6 to 1.7)	−0.086	0.662
		Health-related QoL (EQ-5d-5L) per 0.1 point increase	0.041 (−0.105 to 0.187)	4.2 (−10.0 to 20.6)	0.125	0.571
		PI-NRS	0.024 (−0.092 to 0.139)	2.4 (−8.8 to 14.9)	0.091	0.677
		HADS_depression	0.019 (−0.055 to 0.094)	1.9 (−5.4 to 9.9)	0.102	0.602
Productivity loss	156					
Pain impact group (NRS ≤ 6)	69	Age	−0.013 (−0.051 to 0.025)	−1.3 (−5.0 to 2.5)	−0.078	0.487
		Health-related QoL (EQ-5d-5L) per 0.1 point increase	−0.535 (−0.855 to −0.215)	−41.4 (−57.5 to −19.3)	−0.415	0.001
		HADS_anxiety	−0.033 (−0.168 to 0.102)	−3.3 (−15.5 to 10.7)	−0.062	0.630
		HADS_depression	0.081 (−0.090 to 0.251)	8.4 (−8.6 to 28.5)	0.132	0.347
Pain impact group (NRS ≥ 7)	87	Age	−0.012 (−0.035 to 0.012)	−1.2 (−3.4 to 1.2)	−0.109	0.323
		Health-related QoL (EQ-5d-5L) per 0.1 point increase	−0.040 (−0.195 to 0.116)	−3.9 (−17.7 to 12.3)	−0.057	0.612
		HADS_anxiety	−0.059 (−0.139 to 0.021)	−5.7 (−13.0 to 2.1)	−0.182	0.146
		HADS_depression	0.017 (−0.069 to 0.102)	−1.7 (−6.7 to 10.7)	0.048	0.700
Societal costs	156					
Pain impact group (NRS ≤ 6)	69	Age	−0.011 (−0.025 to 0.002)	1.1 (−2.5 to 0.2)	−0.191	0.089
		Health-related QoL (EQ-5d-5L) per 0.1 point increase	−0.158 (−0.270 to −0.046)	−14.6 (−23.7 to −4.5)	−0.348	0.006
		HADS_anxiety	0.007 (−0.040 to 0.054)	0.7 (−3.9 to 5.5)	0.038	0.769
		HADS_depression	0.025 (−0.035 to 0.084)	2.5 (−3.4 to 8.8)	0.115	0.409
Pain impact group (NRS ≥ 7)	87	Age	−0.001 (−0.009 to 0.007)	−0.1 (−0.9 to 0.7)	−0.023	0.832
		Health-related QoL (EQ-5d-5L) per 0.1 point increase	−0.059 (−0.114 to −0.005)	−5.8 (−10.8 to −0.5)	−0.243	0.031
		HADS_anxiety	−0.021 (−0.049 to 0.007)	−2.0 (−4.8 to 0.7)	−0.186	0.132
		HADS_depression	0.009 (−0.021 to 0.038)	0.6 (−2.1 to 3.9)	0.072	0.556

IA, interaction variable; HADS, Hospital Anxiety and Depression Scale; QoL, Quality of Life; EQ-5D-5L, EuroQol 5D Questionnaire; NRS, Numerical Rating Scale.

## 4. Discussion

To the best of our knowledge, this is the first study examining the healthcare and societal costs of clinically referred patients with confirmed SFN in the Netherlands. The total healthcare costs to Dutch society for the SFN adult population is estimated to be almost €30 million annually, which is approximately 0.03% of the total healthcare expenditure in the Netherlands in 2020 (€106 billion; Central Bureau of Statistics 2020). Overall, health-related QoL was statistically significant associated with SFN patient and family, productivity, and societal costs.

Previous COI of confirmed SFN has not been performed. A cost study of idiopathic painful neuropathy with SFN involvement has been conducted, however,^27^ which allows us to compare our results with previous research. Demographic and clinical characteristics of the 2 study populations were similar, but the study population in the previous study^27^ was insufficiently defined due to inadequate diagnosis of SFN. Therefore, the results may not be a representative for the SFN population. In addition, data in the previous study was collected over a period of 6 months, which is inconsistent with data collection guidelines for costs studies and may have led to an underestimation or overestimation of costs.^28^

Total healthcare and patient and family costs (direct costs) of SFN in our study were lower (€5690), and severe pain was associated with statistically significant higher costs. In the previous study,^27^ only the direct costs of the mild and moderate pain severity groups were statistically significant higher.

The main contributors to the healthcare costs of SFN in our study were medical specialist care and active medical treatment and were different to main contributors identified in the previous study (prescription drugs and out-of-pocket medical costs).^27^ In our SFN study, severe pain was associated with statistically significant higher costs of medical specialist care and active medical treatment, whereas in the previous study, no association was found.^27^

The total productivity costs (indirect costs) of the 2 study populations were similar (approx. €12,000),^27^ and the costs of the severe pain group were significantly higher compared with the mild pain group. In the previous study, the indirect costs of the pain severity groups were not statistically significant higher.^27^

The proportion of patients with SFN in paid employment (47%), who were retired (15%) or disabled (21%) in our study was different compared with the previous study (16%, 49%, and 23%, respectively).^27^

The main contributor to the high costs of productivity loss of SFN in our study was the cost of lost hours due to being limited in performing daily household tasks, which was significantly higher in the severe pain group compared with the mild pain group. In the cost study of idiopathic painful neuropathy with SFN involvement was the main contributor costs of disability, with no association found.^27^

Our average SFN healthcare costs are comparable with the UK healthcare costs of painful diabetic peripheral neuropathy^7^ and the Dutch healthcare costs of fibromyalgia.^34^ Furthermore, the use of SFN pain treatments makes up 28% of the SFN healthcare costs, which is comparable with chronic neuropathic pain treatments in other academic pain centers.^31^ Neuropathic pain is associated with lower health utility scores, and the EQ-5D utility score of our SFN population (0.59) is comparable with diabetic neuropathy (0.61)^13^ and fibromyalgia (0.54).^16^ Comorbidities such as anxiety and depression have a negative effect on Qol in patients with chronic peripheral neuropathic pain,^26^ and our study results showed that severe pain was associated with higher depression scores. Health-related Qol is highly correlated with morbidity, mortality, healthcare, and societal costs,^13^ and in our study, we used the EQ-5D as a generic instrument to measure health-related Qol. The derived utility scores can also be used in a planned cost-effectiveness study.^10^

Regarding the SFN patient and family costs, over-the-counter-medication (eg, nutraceuticals) was often used in addition to the prescribed medications. Nutraceuticals, such as N-palmitoylethanolamide (PEA)^24^ and vitamin D^23^ are increasingly used^1^ and may play a role in neuropathic pain treatment, but more scientific evidence is needed on their effectiveness.

The leading factor of SFN productivity costs was the limitation in performing daily household tasks due to painful SFN. Important for estimating the COI of SFN is including replacement costs for patients' not performed daily household activities. That is, valuing lost productivity hours from unpaid employment activities, which should not be limited to activities actually taken over by informal care givers.^19^ Productivity costs in patients with a NRS ≤ 6 were associated with higher pain impact on daily life and lower health-related QoL. Our results observed a 41% reduction in productivity costs per 0.1 point increase on the EQ-5D utility score.

This study's contribution to the literature is in the detailed insight it provides into the societal COI of patients with confirmed SFN in the Netherlands. This study is based on daily practice data of patients with confirmed SFN, and we were able to investigate a number of associations between costs and patient characteristics.

A limitation of our study was that tertiary care patients were included, who may experience more severe SFN symptoms than patients seeking help in primary or secondary care. However, our sample is a representative for most of the total SFN prevalence figure for 3 reasons: Our study population is comparable with (1) the mean age, the percentage male–female ratio, duration of SFN complaints, and mean average pain of a Swiss study population with SFN,^3^ (2) the healthcare cost study from the US,^27^ and (3) the inclusion of a considerable number of patients with mild and moderate complaints (n = 69). Furthermore, because only older prevalence rates were available while rates are likely increasing due to increased global recognition,^21,25^ this could have led to an underestimation of COI in our study. Another issue may be that costs of absenteeism made in the quarter before registration on the waiting list can be prone to recall bias, because the optimum recall period for loss of productivity is 1 month.^28^ However, the optimal recall period for healthcare, patient, and family costs is different (3 months). Therefore, the recall period of all costs was equalized to 3 months. Finally, the use of the friction cost method for calculating costs of absenteeism may be considered conservative and leads to lower estimates than other methods, such as the human capital method.^19^ However, the friction cost method is in accordance with the Dutch cost guideline.

## 5. Conclusions

An actual cost estimation of patients with confirmed SFN from a Dutch healthcare and societal perspective was provided, with medical specialist care, active medical treatment, and productivity costs identified as the main contributors to these costs. Health-related QoL was the leading association on the high COI of patients with SFN. Future cost-effectiveness studies may explore the impact of therapeutic options for improving the health-related Qol of patients with SFN on the COI of the SFN population.

## Conflict of interest statement

MG, YVEH, LB, and CMLGB have nothing to disclose. JGJH reports a grant from the Prinses Beatrix Spierfonds (W.OK17-09 and W.TR22-01), outside the submitted work. ISJM reports grants from Talecris Talents Program/Perinoms study, grants from GBSjCIDP Foundation International, grants from Prinses Beatrix Fonds, grants from European Union seventh Framework Program, other grants from Steering committee members for various studies, outside the submitted work; serves on the editorial board of the Journal of Peripheral Nervous System, is a member of the Inflammatory Neuropathy Consortium (INC) and member of the Peripheral Nerve Society. MAJ reports grants from European Union's Horizon 2020 and ZonMw, other from participation in the research panel of the Medical Research Council in the United Kingdom. CGF reports grants from European Union's Horizon 2020 (grant no. 721841), grants from Prinses Beatrix Spierfonds, grants from Grifols and Lamepro, other from Steering committees/advisory board for studies of Biogen/Convergence and Vertex, outside the submitted work.

## References

[1] Abdelrahman KM Hackshaw KV. Nutritional supplements for the treatment of neuropathic pain. Biomedicines 2021;9:10. 10.3390/biomedicines9060674 PMC8231824 34199290

[2] Bakkers M Faber CG Hoeijmakers JG Lauria G Merkies IS. Small fibers, large impact: quality of life in small-fiber neuropathy. Muscle Nerve 2014;49:329–36. 23716362 10.1002/mus.23910

[3] Bitzi LM Lehnick D Wilder-Smith EP. Small fiber neuropathy: Swiss cohort characterization. Muscle Nerve 2021;64:293–300. 34075618 10.1002/mus.27340 PMC8453953

[4] Bouwmans C Hakkaart-van Roijen L Koopmanschap M Krol M Severens H Brouwer W. Manual iMTA medical cost questionnaire (iMCQ) [in Dutch: Handleiding iMTA medical cost questionnaire (iMCQ)]. Rotterdam, the Netherlands: Institute for Medical Technology Assessment, 2013.

[5] Bouwmans C Krol M Severens H Koopmanschap M Brouwer W Hakkaart-van Roijen L. The iMTA productivity cost questionnaire: a standardized instrument for measuring and valuing health-related productivity losses. Value Health 2015;18:753–8. 26409601 10.1016/j.jval.2015.05.009

[6] Centraal Bureau voor de Statistiek. Consumentenprijzen. StatLine. Available at: https://opendatacbsnl/statline/#/CBS/nl/dataset/83131ned/table?fromstatweb. Accessed August 31, 2020.

[7] Currie CJ Poole CD Woehl A Morgan CL Cawley S Rousculp MD Covington MT Peters JR. The financial costs of healthcare treatment for people with Type 1 or Type 2 diabetes in the UK with particular reference to differing severity of peripheral neuropathy. Diabetic Med 2007;24:187–94. 17257282 10.1111/j.1464-5491.2006.02057.x

[8] de Greef BTA Hoeijmakers JGJ Gorissen-Brouwers CML Geerts M Faber CG Merkies ISJ. Associated conditions in small fiber neuropathy—a large cohort study and review of the literature. Eur J Neurol 2018;25:348–55. 29112785 10.1111/ene.13508 PMC5814938

[9] de Munter L Geraerds A de Jongh MAC van der Vlegel M Steyerberg EW Haagsma JA Polinder S. Prognostic factors for medical and productivity costs, and return to work after trauma. PLoS One 2020;15:e0230641. 32210472 10.1371/journal.pone.0230641 PMC7094860

[10] Detsky AS Laupacis A. Relevance of cost-effectiveness analysis to clinicians and policy makers. JAMA 2007;298:221–4. 17622605 10.1001/jama.298.2.221

[11] Devigili G Cazzato D Lauria G. Clinical diagnosis and management of small fiber neuropathy: an update on best practice. Expert Rev Neurotherapeutics 2020;20:967–80. 10.1080/14737175.2020.1794825 32654574

[12] Devigili G Rinaldo S Lombardi R Cazzato D Marchi M Salvi E Eleopra R Lauria G. Diagnostic criteria for small fibre neuropathy in clinical practice and research. Brain 2019;142:3728–36. 31665231 10.1093/brain/awz333 PMC6906595

[13] Doth AH Hansson PT Jensen MP Taylor RS. The burden of neuropathic pain: a systematic review and meta-analysis of health utilities. PAIN 2010;149:338–44. 20227832 10.1016/j.pain.2010.02.034

[14] Farrar JT Young JP Jr LaMoreaux L Werth JL Poole RM. Clinical importance of changes in chronic pain intensity measured on an 11-point numerical pain rating scale. PAIN 2001;94:149–58. 11690728 10.1016/S0304-3959(01)00349-9

[15] Finnerup NB Attal N Haroutounian S McNicol E Baron R Dworkin RH Gilron I Haanpaa M Hansson P Jensen TS Kamerman PR Lund K Moore A Raja SN Rice AS Rowbotham M Sena E Siddall P Smith BH Wallace M. Pharmacotherapy for neuropathic pain in adults: a systematic review and meta-analysis. Lancet Neurol 2015;14:162–73. 25575710 10.1016/S1474-4422(14)70251-0 PMC4493167

[16] Franco KFM Cabral CMN Salvador E Miyamoto GC. Comparison between different health state utility instruments in patients with fibromyalgia. Braz J Phys Ther 2021;25:573–82. 33766462 10.1016/j.bjpt.2021.02.006 PMC8536847

[17] Freeman R Gewandter JS Faber CG Gibbons C Haroutounian S Lauria G Levine T Malik RA Singleton JR Smith AG Bell J Dworkin RH Feldman E Herrmann DN Hoke A Kolb N Mansikka H Oaklander AL Peltier A Polydefkis M Ritt E Russell JW Sainati S Steiner D Treister R Uceyler N. Idiopathic distal sensory polyneuropathy: ACTTION diagnostic criteria. Neurology 2020;95:1005–14. 33055271 10.1212/WNL.0000000000010988 PMC7734920

[18] Gore M Brandenburg NA Dukes E Hoffman DL Tai KS Stacey B. Pain severity in diabetic peripheral neuropathy is associated with patient functioning, symptom levels of anxiety and depression, and sleep. J Pain Symptom Manag 2005;30:374–85. 10.1016/j.jpainsymman.2005.04.009 16256902

[19] Hakkaart-van Roijen L van der Linden N Bouwmans C Kanters T Tan SS. Costing manual: Methodology of costing research and reference prices for economic evaluations in healthcare [in Dutch: Kostenhandleiding: Methodologie van kostenonderzoek en referentieprijzen voor economische evaluaties in de gezondheidszorg]. Rotterdam, the Netherlands: Institute for Medical Technology Assessment, 2015.

[20] Herdman M Gudex C Lloyd A Janssen M Kind P Parkin D Bonsel G Badia X. Development and preliminary testing of the new five-level version of EQ-5D (EQ-5D-5L). Qual Life Res 2011;20:1727–36. 21479777 10.1007/s11136-011-9903-x PMC3220807

[21] Johnson SA Shouman K Shelly S Sandroni P Berini SE Dyck PJB Hoffman EM Mandrekar J Niu Z Lamb CJ Low PA Singer W Mauermann ML Mills J Dubey D Staff NP Klein CJ. Small fiber neuropathy incidence, prevalence, longitudinal impairments, and disability. Neurology 2021;97:e2236–e2247. 34706972 10.1212/WNL.0000000000012894 PMC8641968

[22] Johnston M Pollard B Hennessey P. Construct validation of the hospital anxiety and depression scale with clinical populations. J psychosomatic Res 2000;48:579–84. 10.1016/s0022-3999(00)00102-1 11033377

[23] Lee P Chen R. Vitamin D as an analgesic for patients with type 2 diabetes and neuropathic pain. Arch Intern Med 2008;168:771–2. 18413561 10.1001/archinte.168.7.771

[24] Paladini A Fusco M Cenacchi T Schievano C Piroli A Varrassi G. Palmitoylethanolamide, a special food for medical purposes, in the treatment of chronic pain: a pooled data meta-analysis. Pain Physician 2016;19:11–24. 26815246

[25] Peters MJ Bakkers M Merkies IS Hoeijmakers JG van Raak EP Faber CG. Incidence and prevalence of small-fiber neuropathy: a survey in The Netherlands. Neurology 2013;81:1356–60. 23997150 10.1212/WNL.0b013e3182a8236e

[26] Radat F Margot-Duclot A Attal N. Psychiatric co-morbidities in patients with chronic peripheral neuropathic pain: a multicentre cohort study. Eur J Pain 2013;17:1547–57. 23720357 10.1002/j.1532-2149.2013.00334.x

[27] Schaefer C Mann R Sadosky A Daniel S Parsons B Nalamachu S Stacey BR Tuchman M Anschel A Nieshoff E. Health status, function, productivity, and costs among individuals with idiopathic painful peripheral neuropathy with small fiber involvement in the United States: results from a retrospective chart review and cross-sectional survey. J Med Econ 2014;17:394–407. 24673364 10.3111/13696998.2014.909439

[28] Severens JL Mulder J Laheij RJ Verbeek AL. Precision and accuracy in measuring absence from work as a basis for calculating productivity costs in The Netherlands. Soc Sci Med 2000;51:243–9. 10832571 10.1016/s0277-9536(99)00452-9

[29] Sopacua M Hoeijmakers JGJ Merkies ISJ Lauria G Waxman SG Faber CG. Small-fiber neuropathy: expanding the clinical pain universe. J Peripher Nervous Syst 2019;24:19–33. 10.1111/jns.12298 30569495

[30] Tan SS Bouwmans CA Rutten FF Hakkaart-van Roijen L. Update of the Dutch manual for costing in economic evaluations. Int J Technol Assess Health Care 2012;28:152–8. 22559757 10.1017/S0266462312000062

[31] Tarride JE Moulin DE Lynch M Clark AJ Stitt L Gordon A Morley-Forster PK Nathan H Smyth C Toth C Ware MA. Impact on health-related quality of life and costs of managing chronic neuropathic pain in academic pain centres: results from a one-year prospective observational Canadian study. Pain Res Manage 2015;20:327–33. 10.1155/2015/214873 PMC4676504 26474381

[32] van der Vlegel M Haagsma JA Geraerds A de Munter L de Jongh MAC Polinder S. Health care costs of injury in the older population: a prospective multicentre cohort study in The Netherlands. BMC Geriatr 2020;20:417. 33087050 10.1186/s12877-020-01825-z PMC7576762

[33] Versteegh MM Vermeulen KM Evers SAA de Wit GA Prenger R Stolk EA. Dutch tariff for the five-level version of EQ-5D. Value Health 2016;19:343–52. 27325326 10.1016/j.jval.2016.01.003

[34] Vervoort VM Vriezekolk JE Olde Hartman TC Cats HA van Helmond T van der Laan WH Geenen R van den Ende CH. Cost of illness and illness perceptions in patients with fibromyalgia. Clin Exp Rheumatol 2016;34:S74–82. 26886404

[35] World Medical Association. World Medical Association Declaration of Helsinki: ethical principles for medical research involving human subjects. JAMA 2013;310:2191–4. 24141714 10.1001/jama.2013.281053

[36] Zelman DC Dukes E Brandenburg N Bostrom A Gore M. Identification of cut-points for mild, moderate and severe pain due to diabetic peripheral neuropathy. PAIN 2005;115:29–36. 15836967 10.1016/j.pain.2005.01.028

